# Fluorescent reporters for functional analysis in rice leaves

**DOI:** 10.1002/pld3.188

**Published:** 2020-02-11

**Authors:** Leonie H. Luginbuehl, Sherif El‐Sharnouby, Na Wang, Julian M. Hibberd

**Affiliations:** ^1^ Department of Plant Sciences University of Cambridge Cambridge UK

**Keywords:** fluorescent proteins, microparticle bombardment, rice leaves, stable transformants

## Abstract

Fluorescent reporters have facilitated non‐invasive imaging in multiple plant species and thus allowed the analysis of processes ranging from gene expression and protein localization to cellular patterning. However, in rice, a globally important crop and model species, there are relatively few reports of fluorescent proteins being used in leaves. Fluorescence imaging is particularly difficult in the rice leaf blade, likely due to a high degree of light scattering in this tissue. To address this, we investigated approaches to improve deep imaging in mature rice leaf blades. We found that ClearSee treatment, which has previously been used to visualize fluorescent reporters in whole tissues of plants, led to improved imaging in rice. Removing epidermal and subtending mesophyll cell layers was faster than ClearSee and also reduced light scattering such that imaging of fluorescent proteins in deeper leaf layers was possible. To expand the range of fluorescent proteins suitable for imaging in rice, we screened twelve whose spectral profiles spanned most of the visible spectrum. This identified five proteins (mTurquoise2, mNeonGreen, mClover3, mKOκ, and tdTomato) that are robustly expressed and detectable in mesophyll cells of stably transformed plants. Using microparticle bombardment, we show that mTurquoise2 and mNeonGreen can be used for simultaneous multicolor imaging of different subcellular compartments. Overall, we conclude that mTurquoise2, mNeonGreen, mClover3, mKOκ, and tdTomato are suitable for high‐resolution live imaging of rice leaves, both after transient and stable transformation. Along with the rapid microparticle bombardment method, which allows transient transformation of major cell types in the leaf blade, these fluorescent reporters should greatly facilitate the analysis of gene expression and cell biology in rice.

## 1 INTRODUCTION

The availability of fluorescent proteins has advanced our understanding of biological processes such as membrane trafficking and the subcellular localization of proteins, the spatial and temporal regulation of gene expression, and the dynamics of cellular patterning and organ development. Unlike other reporters, including β‐glucuronidase (GUS) and luciferase, fluorescent proteins allow the visualization and relative quantification of proteins in live tissue without the need to add a substrate or cofactor (Hanson & Köhler, 2001; Haseloff, 1998; Jefferson, Kavanagh, & Bevan, 1987; Luehrsen, Wet, & Walbot, 1992). In addition, the development of a wide range of color variants based on the original green fluorescent protein (GFP) from *Aequorea victoria* and the discovery of new fluorescent proteins from other species have enabled multicolor imaging and the simultaneous visualization of multiple processes in the same tissue, cell, or subcellular compartment (Prasher, Eckenrode, Ward, Prendergast, & Cormier, 1992; Shaner, Steinbach, & Tsien, 2005). Fluorescent reporters are also an important component of many experimental tools, such as protein tagging for chromatin immunoprecipitation (De Folter, Urbanus, Zuijlen, Kaufmann, & Angenent, 2007) and fluorescence‐activated cell sorting (Birnbaum et al., 2005).

In many plant species, imaging of fluorescent proteins is commonly reported for all major plant organs (Bellucci, Marchis, Mannucci, Bock, & Arcioni, 2005; Cutler, Ehrhardt, Griffitts, & Somerville, 2000; Mohanty et al., 2009). However, in rice, while they are routinely used for analysis in roots and other non‐green tissues (Abiko et al., 2013; Chu et al., 2018; Kim, Yuan, Cilia, Khalfan‐Jagani, & Jackson, 2002; Kobae & Hata, 2010; Wu et al., 2016), there are fewer examples of fluorescent reporters being used in leaves (Dangol, Singh, Chen, & Jwa, 2017; Johnson et al., 2005; Meng, Hsiao, Gao, Jiang, & Chye, 2014; Reynoso et al., 2018; Wang, Zhao, Fu, Wang, & Jiang, 2018; Wu et al., 2016). In fact, most of these reports show fluorescent protein expression in the leaf sheath (Dangol et al., 2017; Meng et al., 2014; Wang et al., 2018; Wu et al., 2016) and few show expression in the leaf blade (Johnson et al., 2005; Reynoso et al., 2018). Moreover, in the leaf blade, expression has only been reported in cells at the leaf surface and not in deeper leaf layers (Johnson et al., 2005; Reynoso et al., 2018). This is likely due to the high degree of light scattering arising from the waxy cuticle of the rice leaf blade, which impedes high‐resolution fluorescent protein visualization in deeper layers (Clark & Lister, 1975; Qin et al., 2011). In recent years, however, several methods have been developed to clear tissue for whole‐organ or whole‐plant imaging (Kurihara, Mizuta, Sato, & Higashiyama, 2015; Palmer et al., 2015; Timmers, 2016). Among these, the ClearSee protocol enables deep imaging in Arabidopsis whole leaves while at the same time maintaining the stability and thus enabling the visualization of fluorescent proteins (Kurihara et al., 2015).

In addition to the difficulties associated with deep tissue imaging of rice leaves, to our knowledge, only EGFP (Reynoso et al., 2018), G3GFP, EYFP, mRFP1 (Dangol et al., 2017), and DsRed (Meng et al., 2014) have been reported to be expressed in rice leaves. Thus, available tools for fluorescence imaging in rice leaves are currently limited, hindering the analysis of biological processes such as leaf development, photosynthesis, and chloroplast biogenesis.

The use of fluorescent reporters relies on the ability to stably or transiently transform plant tissue with the required expression vectors. Although rice is widely used as a model due to its relatively small and sequenced genome (Goff et al., 2002; Matsumoto et al., 2005), the availability of a range of genetic resources, and the high level of genomic synteny to other cereals (Bennetzen & Freeling, 1997), stable transformation is labor‐intensive and time consuming (Hiei & Komari, 2008; Supartana et al., 2005; Toki et al., 2006). Thus, heterologous expression systems such as *Arabidopsis thaliana* and *Nicotiana benthamiana* or transient protoplast assays are routinely used to investigate the regulation of rice genes as well as the function and subcellular localization of rice proteins (Kitajima et al., 2009; Lam et al., 2007; Zhang et al., 2011). Although less time consuming, these approaches do not allow analysis in cells of intact rice tissues such as fully developed leaves. Transient transformation by microparticle bombardment is an alternative approach that has successfully been employed to transform individual cells in whole leaves of several plant species (Brown et al., 2011; Reyna‐Llorens et al., 2018)*.* For rice, even though microparticle bombardment has been used for transient expression in the root and epidermal cells of the leaf sheath (Dangol et al., 2017; Meng et al., 2014; Wang et al., 2018), to our knowledge, there are few reports of successful microparticle bombardment of the mature rice leaf blade (Jia, Mcadams, Bryan, Hershey, & Valent, 2000).

Here, we tested and optimized tools and methodology for the expression and imaging of fluorescent proteins in all layers of the rice leaf blade. We tested two methods for deep imaging in rice leaves, clearing using the ClearSee protocol (Kurihara et al., 2015) and scraping the leaf surface, and found that both methods improve visualization of fluorescent reporters in deeper leaf layers. To expand the range of fluorescent proteins available for imaging in rice, we generated stable transformants expressing twelve reporters with distinct spectral characteristics and tested the extent to which each was detectable in the rice leaf blade. From this analysis, we identified five fluorescent proteins that are robustly detectable in rice mesophyll cells. Lastly, microparticle bombardment was optimized to provide a fast and simple method to transiently transform different cell types in the rice leaf blade. Using fluorescent reporters in stable lines or after transient transformation should facilitate our ability to analyze biological processes such as gene expression, protein subcellular localization and protein–protein interactions in rice, a globally important crop and model species.

## 2 MATERIALS AND METHODS

### 2.1 Cloning and construct design

Fluorescent proteins chosen as candidate reporters were selected for screening primarily based on their brightness (Figure S1), but also taking into consideration parameters such as resistance to photobleaching, thermostability, rapid maturation time, and minimal spectral overlap with chlorophyll autofluorescence (https://www.fpbase.org; Lambert, 2019). Coding sequences of twelve fluorescent proteins were obtained from the following sources: mTurquoise2 (Goedhart et al., 2012), mTFP1 [GenBank accession number DQ676819.1, (Ai, Henderson, Remington, & Campbell, 2006)], mNeonGreen [GenBank accession number KC295282.1, (Shaner et al., 2013)], mClover3 (Bajar et al., 2016), mCitrine (Griesbeck, Baird, Campbell, Zacharias, & Tsien, 2001), mYPet [the monomerizing mutation A206K was introduced into the YPet sequence (Nguyen & Daugherty, 2005) to generate mYPet], mKOκ [7 amino acid mutations (K49E, P70V, K185E, K188E, S192D, S196G, and L210Q) were introduced into the mKO sequence with GenBank accession number AB128821.1 to generate mKOκ, (Tsutsui, Karasawa, Okamura, & Miyawaki, 2008)], tdTomato (Shaner et al., 2004), TagRFP‐T [GenBank accession number EU582019.1, (Shaner et al., 2008)], mRuby3 (Bajar et al., 2016), mKate2 (Shcherbo et al., 2009), and mCardinal (Chu et al., 2014). Prior to synthesis for Golden Gate cloning (Engler, Gruetzner, Kandzia, & Marillonnet, 2009; Weber, Engler, Gruetzner, Werner, & Marillonnet, 2011), coding sequences were codon‐optimized for rice using the codon optimization tool by Integrated DNA Technologies, domesticated for the Golden Gate cloning system, and modified such that the codon encoding valine at position 2 of each open reading frame was mutated to code for an alanine (GCG) to achieve a partial match to the Kozak consensus sequence for monocotyledons (Gupta, Rangan, Ramesh, & Gupta, 2016; Joshi, Zhou, Huang, & Chiang, 1997; Nakagawa, Niimura, Gojobori, Tanaka, & Miura, 2008). Where relevant, the GFP cryptic intron (Haseloff, Siemering, Prasher, & Hodge, 1997) and potential intron sites identified by NetPlantGene Server (Hebsgaard et al., 1996) were neutralized. All fluorescent protein sequences are listed in Table S1. Each fluorescent protein module was synthesized as an N‐terminal tag and fused at the C‐terminal end with a GGAGSGAGG amino acid linker to a codon‐optimized N7 nuclear localization signal peptide (Cutler et al., 2000). These Level 0 modules were assembled together with the maize *PEPC* promoter (Matsuoka, Kyozuka, Shimamoto, & Kano‐Murakami, 1994) and *Cauliflower Mosaic Virus 35S* terminator into Level 1 modules, then combined with the hygromycin resistance gene into the Level 2 binary vector pICSL4723. The Level 0 modules for the robustly detectable fluorescent proteins mTurquoise2, mNeonGreen, mClover3, mKOκ, and tdTomato have been made available through Addgene.

For transient expression by microparticle bombardment, the chloroplast targeting sequence *RC2* (Shen et al., 2017) and sequence for the plasma membrane‐localized protein *OsPIP2.1* (Dangol et al., 2017) were domesticated for the Golden Gate cloning system and synthesized. Level 1 modules were assembled through ligation of the *ZmUBIQUITIN* or *OsACT1* promoter sequence (Cornejo, Luth, Blankenship, Anderson, & Blechl, 1993; McElroy, Zhang, Cao, & Wu, 1990); the coding sequence for mTurquoise2 or mNeonGreen; the nuclear, chloroplast, or plasma membrane targeting sequence; and the *NOS* or *CaMV35S* terminator sequence. Microparticle bombardment was performed using the Level 1 modules assembled into the Level 2 binary vector pICSL4723. Assembly of the *GUS* reporter construct used for microparticle bombardment has been described previously (Brown et al., 2011).

### 2.2 Stable transformation of rice

*Oryza sativa* spp. *japonica* cultivar Kitaake was transformed using *Agrobacterium tumefaciens* as described previously (Hiei & Komari, 2008) with several modifications. Seeds were de‐husked and sterilized with 10% (v/v) bleach for 15 min. Seeds were placed on nutrient broth (NB) callus induction medium containing 2 mg/L 2,4‐dichlorophenoxyacetic acid for 3 to 4 weeks at 28°C in the dark. Actively growing calli were co‐incubated with transformed *A. tumefaciens* strain LBA4404 in NB inoculation medium containing 40 μg/ml acetosyringone for 3 days at 22°C in the dark. Calli were transferred to NB recovery medium containing 300 mg/L timentin for 1 week at 28°C in the dark then to NB selection medium containing 35 mg/L hygromycin B for 4 weeks at 28°C in the dark. Proliferating calli were subsequently transferred to NB regeneration medium (containing 100 mg/L myo‐inositol, 2 mg/L kinetin, 0.2 mg/L 1‐naphthaleneacetic acid, and 0.8 mg/L 6‐benzylaminopurine) for 4 weeks at 28°C in the light. Plantlets were then transferred to NB rooting medium containing 0.1 mg/L 1‐naphthaleneacetic acid and incubated in Magenta pots for 2 weeks at 28°C in the light. Finally, plants were transferred to a 1:1 mixture of topsoil and sand and grown in a greenhouse under natural light supplemented with a minimum light intensity of 390 μmol m^−2^ s^−1^, with 60% humidity, temperatures of 28°C and 23°C during the day and night, respectively, and a photoperiod of 12‐hr light and 12‐hr dark.

### 2.3 Microparticle bombardment

*Oryza sativa *spp.* japonica* cultivar Kitaake was used for transient transformation of leaf and root cells by microparticle bombardment. Seeds were de‐husked prior to sterilization in 5% (v/v) bleach containing 0.5% (v/v) Tween‐20 for 30 min. Seeds were placed on half strength Murashige and Skoog (MS) medium containing 0.8% (w/v) agar and 1% (w/v) sucrose, and germinated in the dark at 28°C. Seedlings were grown in the dark at 28°C for 12 to 16 days and leaf 2 (of 12‐day‐old seedlings), leaf 3 (of 16‐day‐old seedlings), or roots (of 12‐day‐old seedlings) used for microparticle bombardment.

Plasmid DNA was bound to 1 μm DNAdel™ Gold Carrier Particles (Seashell Technology) according to the manufacturer's instructions. For each expression vector, 5 μg DNA was bound to 1.5 mg particles prior to resuspension in 70 μl 100% (v/v) ethanol. Seven microliters of the particle suspension was transferred to plastic macrocarriers (Bio‐Rad) and allowed to dry completely at room temperature. For each transformation, leaf blades or roots were cut into 4‐cm‐long pieces, placed on 0.8% (w/v) water agar and bombarded three times with a Bio‐Rad PDS‐1000/He particle delivery system using 1,800 psi (for leaves) or 1,550 psi (for roots) rupture disks (Bio‐Rad). For transformation of leaf cells, the abaxial side of the leaf blade was bombarded. After bombardment, leaf blades or roots were flooded with half strength MS solution containing 1% (w/v) sucrose and incubated in the dark (or continuous light for chloroplast‐targeted proteins) at 28°C for 24 hr before analysis by confocal scanning laser microscopy or staining for GUS.

### 2.4 GUS staining

To visualize GUS activity in transiently transformed cells, leaf blades or roots were submerged in GUS staining solution [0.1 M Na_2_HPO_4_ pH 7.0, 0.5 mM K ferricyanide, 0.5 mM K ferrocyanide, 0.05% (v/v) Triton X‐100, 10 mM Na_2_EDTA pH 8.0, 1 mM X‐Gluc (5‐bromo‐4‐chloro‐3‐indolyl‐beta‐D‐glucuronide)] 24 hr after microparticle bombardment. For leaf blades, a vacuum was applied four times for 2 min each time to submerge the tissue. Plant material was incubated in GUS staining solution for 6 to 24 hr at 37°C in the dark and fixed in a 3:1 solution of ethanol and acetic acid for 30 min at room temperature. For leaf blades, chlorophyll was cleared with 70% (v/v) ethanol, followed by incubation in 5% (w/v) NaOH for 2 hr at 37°C. Bright field images of GUS‐stained plant tissue were obtained with an Olympus BX41 light microscope.

### 2.5 Tissue preparation and confocal imaging

Prior to confocal imaging, leaf tissue was prepared either by scraping the leaf surface or clearing the leaves using the ClearSee protocol to enable visualization of deeper tissue layers.

The adaxial side of 4‐cm‐long segments of the leaf blade from mature rice leaves was scraped two to three times with a sharp razor blade, transferred to phosphate‐buffered saline (PBS) to avoid drying and then mounted on a microscope slide with the scraped surface facing upwards. The ClearSee protocol was performed as previously described (Kurihara et al., 2015) with several modifications. Leaf blades were cut into 5‐mm‐long segments and fixed in 4% (w/v) formaldehyde solution (freshly prepared from paraformaldehyde and containing 0.1% Silwet L‐77) for 1.5 to 3 hr under vacuum at room temperature. For the imaging of mClover3, we found that scraping the leaf surface prior to fixation was important for the effective preservation of mClover3 fluorescence, and even though fixation for 1.5 hr was sufficient for mTurquoise2, mKOκ, and tdTomato, fixation for 3 hr was required for mClover3. In contrast, neither scraping the leaf surface prior to fixation nor increasing the duration of fixation to 8 hr or overnight adequately preserved mNeonGreen fluorescence. During fixation, vacuum was initially released and reapplied five times, then every 15 min thereafter. After washing the leaves twice with PBS for 1 min each, they were cleared with ClearSee solution [10% (w/v) xylitol, 15% (w/v) sodium deoxycholate, and 25% (w/v) urea] for 4 days to 4 weeks with gentle agitation at room temperature and ClearSee solution replaced every 1 to 3 days. As reported for Arabidopsis leaves (Kurihara et al., 2015), 4 days of clearing was found to be sufficient for young rice leaves.

Confocal imaging was performed on a Leica TCS SP8 X using a 10X air objective (HC PL APO CS2 10X 0.4 Dry) with or without optical zoom, and hybrid detectors for fluorescent protein and chlorophyll autofluorescence detection. The excitation and emission spectra of the fluorescent proteins are available at (https://www.fpbase.org; Lambert, 2019), and the following excitation (Ex) and emission (Em) wavelengths were used for imaging: mTurquoise2 (Ex = 442, Em = 471–481), mTFP1 (Ex = 442, Em = 477–517), mNeonGreen (Ex = 506, Em = 512–522), mClover3 (Ex = 506, Em = 513–523), mCitrine (Ex = 516, Em = 524–534), mYPet (Ex = 517, Em = 525–535), mKOκ (Ex = 551, Em = 558–568), tdTomato (Ex = 554, Em = 576–586), TagRFP‐T (Ex = 555, Em = 579–589), mRuby3 (Ex = 558, Em = 587–597), mKate2 (Ex = 588, Em = 628–638), mCardinal (Ex = 604, Em = 654–664), chlorophyll autofluorescence (Ex = 488, Em = 672–692).

## 3 RESULTS

### 3.1 Identification of fluorescent reporters that are stably expressed and detectable in the rice leaf blade

**Figure 1:**
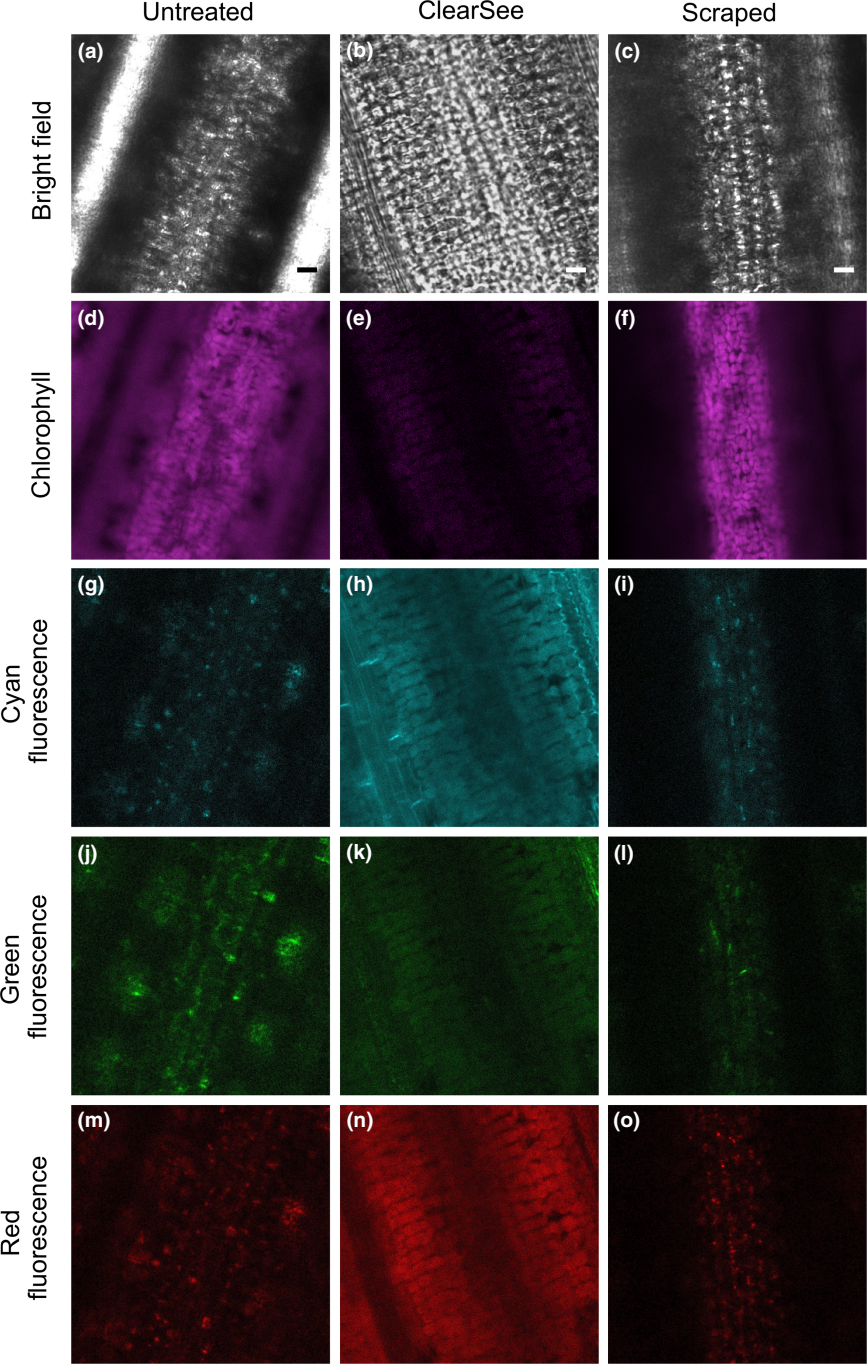
Deep tissue imaging of the rice leaf blade. Representative bright field and confocal images of the mesophyll layer from untreated wild‐type leaves (a,d,g,j,m). Note the lack of clarity in the bright field (a) and chlorophyll autofluorescence (d) images due to light scattering. Untreated leaves also contain structures that emit considerable autofluorescence in the cyan, green and red channels (g,j,m). Treatment with ClearSee improved definition of bright field images (b). Leaf scraping reduced blurring in bright field (c) and chlorophyll autofluorescence (f) images, but did not remove signal coming from the autofluorescent structures detected in the cyan, green, and red channels (i,l,o). A gain of 250 was used to acquire all images in the cyan, green, and red channels, except for (h), where a reduced gain of 100 was used due to high levels of background signal in leaves treated with ClearSee. Note that although ClearSee largely reduces signal from autofluorescent structures including chloroplasts, there tends to be an increase in the homogeneous background signal. Scale bars represent 10 μm

Fluorescence imaging of rice leaves is impaired by high levels of light scattering arising from the leaf surface, which impedes in‐focus, high‐resolution imaging of deeper leaf layers such as the mesophyll cells (Clark & Lister, 1975; Qin et al., 2011; Figure 1a,d). We tested whether the ClearSee protocol that enables deep imaging of Arabidopsis leaves (Kurihara et al., 2015) can also be used to improve whole‐leaf imaging in rice. Four days of clearing rice leaves led to improved visualization of different layers of the leaf blade, including mesophyll cells (Figure 1b). While these results suggest that ClearSee is an effective method for deep imaging in rice leaves, the protocol requires fixation followed by several days of clearing. We therefore sought a faster method that allows live imaging of the leaf interior. We manually removed the leaf surface by scraping the adaxial side of each leaf with a razor blade and mounted the leaf with the scraped surface facing upwards to allow unimpeded laser interrogation of the leaf interior. We found that this also enabled in‐focus visualization of mesophyll cells (Figure 1c,f). Bright autofluorescent structures associated with the mesophyll layer were detected in almost all regions of the visible spectrum in both scraped (Figure 1i,l,o) and non‐scraped (Figure 1g,j,m) leaves. Treatment with ClearSee reduced signal from these autofluorescent structures (Figure 1h,k,n). Together, these results suggest that both methods can be used to overcome light scattering for high‐resolution imaging of the leaf interior. While scraping the leaf surface is a quick method for improved live imaging, the ClearSee protocol involves fixation and several days of clearing but has the advantage of reducing signal from autofluorescent structures in deeper tissue layers.

**Figure 2:**
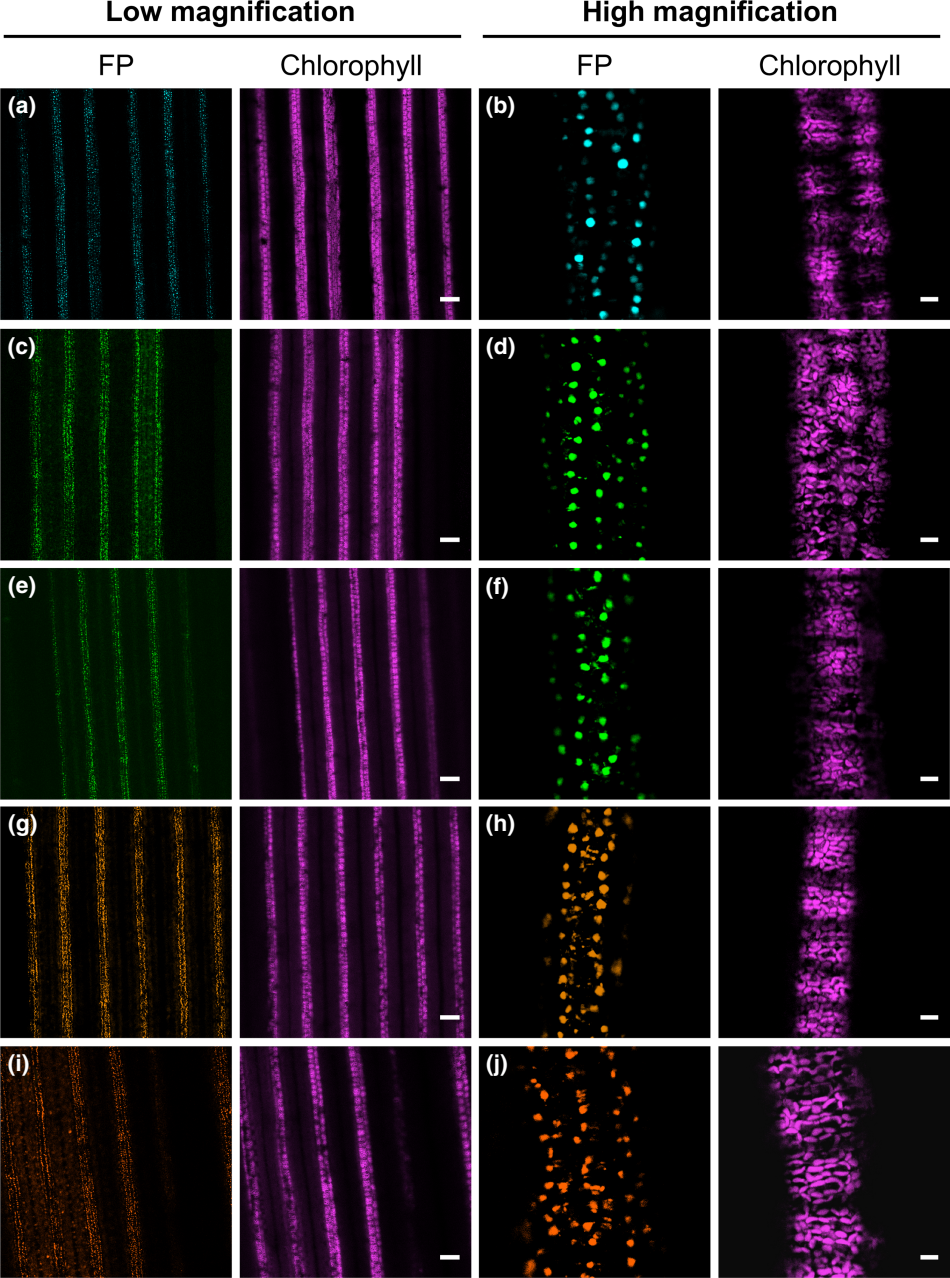
Robustly detectable fluorescent proteins expressed in mesophyll cells of rice leaf blades from stably transformed T_0_ plants. (a,b) Leaves expressing *ZmPEPC_pro_:mTurquoise2‐NLS.* (c,d) Leaves expressing *ZmPEPC_pro_:mNeonGreen‐NLS.* (e,f) Leaves expressing *ZmPEPC_pro_:mClover3‐NLS.* (g,h) Leaves expressing *ZmPEPC_pro_:mKOκ‐NLS.* (i,j) Leaves expressing *ZmPEPC_pro_:tdTomato‐NLS*. FP; fluorescent protein. Scale bars represent 100 μm and 10 μm for low and high magnification images, respectively

Over the years, a large number of fluorescent protein variants with different excitation and emission spectra have been developed and successfully used in many plant species. Being able to combine these different fluorescent reporters allows the co‐visualization of multiple compartments or biological processes in the same cell (DeBlasio, Sylvester, & Jackson, 2010; Shaner et al., 2005). To our knowledge, however, only GFP has been visualized in the rice leaf blade (Johnson et al., 2005; Reynoso et al., 2018). To expand the range of fluorescent proteins that can be used for functional analysis in rice leaves, we assessed twelve proteins that emit light from the cyan to far‐red regions of the spectrum for inherent brightness in stably transformed rice plants (Figure S1). To facilitate imaging and clearly distinguish genuine fluorescent protein signal from autofluorescent structures, we placed each reporter under the control of the maize *PEPC* promoter to direct gene expression to mesophyll cells (Matsuoka et al., 1994) and used a nuclear localization signal to specifically label nuclei. We assessed five to nine independent T_0_ lines for each reporter and scraped the leaf surface prior to confocal microscopy for improved visualization. The degree of signal from each protein was variable, but five were robustly detectable despite autofluorescent structures in the mesophyll layer (Figure 2, Figure S2, Table S2). mTurquoise2 generated the clearest signal, but mNeonGreen, mClover3, mKOκ, and tdTomato were also clearly detectable (Figure 2). The remaining fluorescent proteins were either not detectable at all (mTFP1, mRuby3, mKate2, and mCardinal) or only poorly detectable (mCitrine, mYPet, and TagRFP‐T) (Figure S2, Table S2). To confirm that accumulation of mTurquoise2, mNeonGreen, mClover3, mKOκ, and tdTomato was stable in subsequent generations of transgenic rice plants, three independent T_1_ plants for each construct were imaged by confocal microscopy. T_1_ plants showed detectable signal in mesophyll nuclei for all five proteins (Figure S3), indicating that transgene expression and fluorescent protein accumulation are maintained in the next generation of transgenic plants.

We also tested whether the stability of the five fluorescent reporters is maintained after ClearSee treatment. To this end, leaf tissue from transgenic plants was fixed with formaldehyde. After ClearSee treatment, strong fluorescence signal was detectable in mesophyll nuclei of leaves expressing mTurquoise2, mClover3, mKOκ, and tdTomato (Figure S4a‐c,g‐o). However, only weak nuclear signal was detectable in mesophyll cells of rice plants expressing mNeonGreen (Figure S4d‐f), and the signal was restricted to mesophyll cells located at the edge of cut leaves. These results indicate that although the ClearSee conditions used here are not yet optimized for robust visualization of mNeonGreen, they are suitable for the detection of mTurquoise2, mClover3, mKOκ, and tdTomato.

### 3.2 A transient expression system for functional analysis in the rice leaf blade

**Figure 3:**
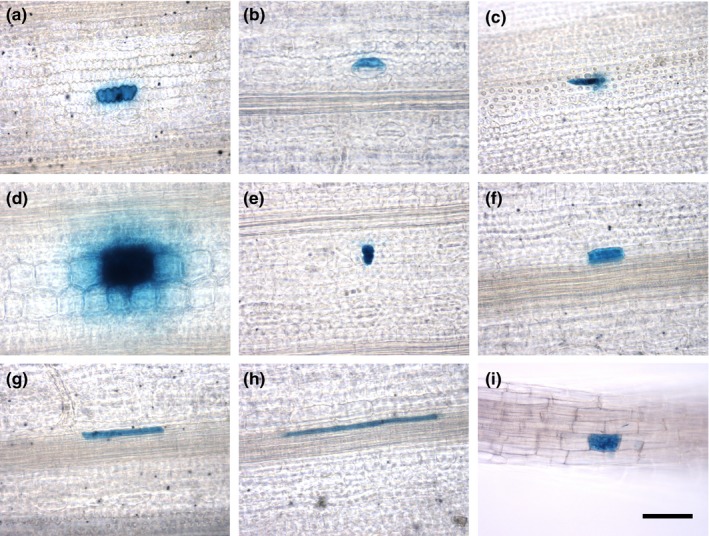
Microparticle bombardment of rice leaf blades and roots. GUS accumulation in leaf and root cells transformed with a *CaMV35S_pro_:GUS* vector using microparticle bombardment. A transformed leaf epidermal (a), guard (b), trichome (c), bulliform (d), mesophyll (e), bundle sheath (f), mestome sheath (g), veinal (h), as well as root (i) cell. All images were taken at the same magnification, and the scale bar represents 50 μm

**Figure 4:**
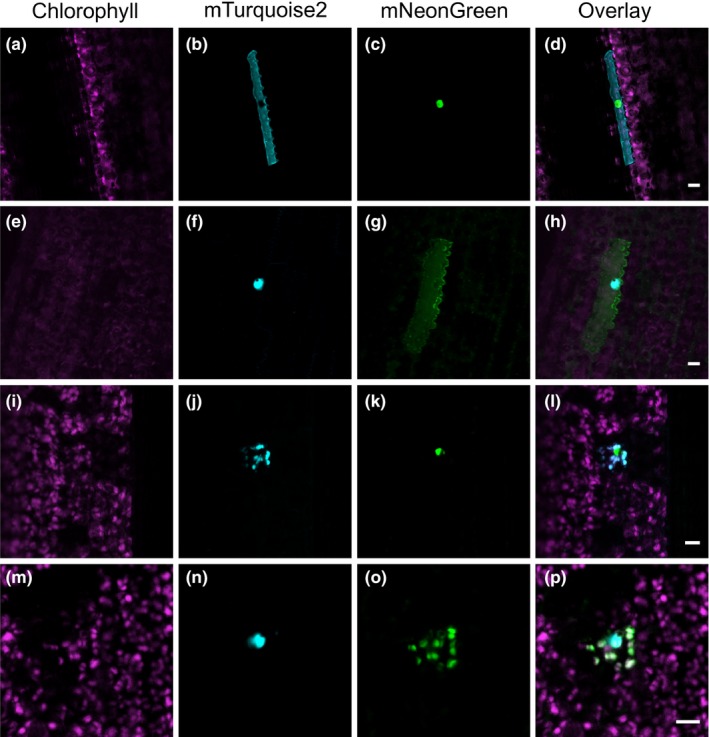
Fluorescent proteins targeted to different cell compartments in transiently transformed cells of the rice leaf blade. (a‐d) Epidermal cell transformed with a construct expressing plasma membrane‐localized mTurquoise2 and nuclear‐localized mNeonGreen. (e‐h) Epidermal cell transformed with a construct expressing nuclear‐localized mTurquoise2 and plasma membrane‐localized mNeonGreen. (i‐l) Mesophyll cell transformed with a construct expressing chloroplast‐localized mTurquoise2 and nuclear‐localized mNeonGreen. (m‐p) Mesophyll cell transformed with a construct expressing nuclear‐localized mTurquoise2 and chloroplast‐localized mNeonGreen. Scale bars represent 10 μm

We next tested whether microparticle bombardment can be used as a fast and simple technique to transiently transform different cell types in the leaf blade of fully developed rice leaves. Epidermal, guard, trichome, bulliform, mesophyll, bundle sheath, mestome sheath, and veinal cells were successfully transformed after bombardment with a *GUS* reporter gene under the control of the constitutively active *CaMV35S* promoter (Figure 3a‐h). On average, 34 ± 18 cells per leaf (*n* = 6 leaves) showed transgene expression, suggesting that microparticle bombardment is an efficient method to transiently transform a range of different cell types from fully expanded rice leaves. We next tested whether we can use microparticle bombardment to fluorescently label different cell compartments simultaneously using the fluorescent reporters identified above. To this end, vectors for the co‐expression of mTurquoise2 and mNeonGreen targeted to either the nucleus (Cutler et al., 2000), plasma membrane (Dangol et al., 2017), or chloroplast (Shen et al., 2017) and driven by constitutive promoters were generated. Bombarding rice leaves with these vectors resulted in transformed cells containing both mTurquoise2 and mNeonGreen targeted to two different cell compartments (Figure 4). Similar to the results with the *GUS* reporter gene, we were able to detect these fluorescent proteins using confocal microscopy in a range of different cell types, including epidermal cells and mesophyll cells (Figure 4). In addition to using microparticle bombardment for rice leaves, we also tested whether tissues such as roots could be transformed using our experimental conditions. We found that bombardment of roots with a *GUS* reporter gene resulted in successfully transformed root cells (Figure 3i). Moreover, the nucleus and plasma membrane of root cells could be simultaneously labeled with mTurquoise2 and mNeonGreen (Figure S5). These results indicate that mTurquoise2 and mNeonGreen are not only suitable for imaging in rice leaves, but are also stably expressed and detectable in rice roots.

## 4 DISCUSSION

Rice is the staple food for almost two thirds of the world's population (Arendt & Zannini, 2013; Gnanamanickam, 2009) and so is one of the most important food crops in the world. If we aim to engineer rice with increased yield, improvements in our understanding of its biology are likely required. Fluorescent reporters greatly facilitate the analysis of biological processes; however, such tools are currently limited for rice leaves. Of the twelve fluorescent proteins that we tested, seven could not be detected reliably under our experimental conditions. Several reasons could account for this. It is possible that codon optimization introduces sequence motifs that repress either transcription or translation, or that it adversely affects mRNA stability or interferes with protein folding and thus disrupts protein function (Hanson & Coller, 2017; Yu et al., 2015; Zhao, Yu, & Liu, 2017). It is also possible that transcripts are silenced in transgenic plants, or that proteins are unstable and degraded. Further analyses are required to test these possibilities. While we cannot completely rule out the possibility that strong autofluorescence has made these proteins particularly challenging to detect, we consider this unlikely for two reasons. First, nuclei are generally minimally autofluorescent and so any stable expression of a nuclear‐localized fluorescent protein should be detectable despite autofluorescence elsewhere in the cell. Second, despite substantial autofluorescence in the green to red regions of the spectrum, the fluorescent proteins mNeonGreen, mClover3, mKOκ, and tdTomato were all robustly detectable.

Although seven of the proteins we tested were not robustly detectable, five were expressed and easily visible in the rice leaf blade. Moreover, these proteins emit light in different regions of the spectrum such that a repertoire of cyan, green, orange, and red fluorescent proteins has been identified. These reporters are therefore complementary to EGFP, which has previously been detected in epidermal and guard cells of the rice leaf blade (Reynoso et al., 2018). mTurquoise2 generated the most clearly detectable signal owing to its brightness and relatively little signal from autofluorescent structures in the cyan region of the spectrum. Although there appears to be more substantial signal from autofluorescent structures in the green to red regions of the spectrum, the fluorescent proteins mNeonGreen, mClover3, mKOκ, and tdTomato were also robustly detectable. Based on these results, we propose that these five fluorescent reporters are suitable for fluorescence imaging and other fluorescence‐based applications, such as protein tagging for immunoprecipitation or fluorescence‐activated cell sorting, in rice leaves. Because the identified proteins emit light in three different spectral ranges (cyan, green, and orange/red), combinations of these reporters can be used to co‐visualize several proteins, or label several cell compartments or cell types, in leaves and other organs of rice.

We have shown that ClearSee treatment is compatible with the use of mTurquoise2, mClover3, mKOκ, and tdTomato in rice leaves. However, for mNeonGreen, nuclear‐localized fluorescence was only poorly detectable after ClearSee treatment. It is possible that the three‐dimensional conformation of mNeonGreen is more susceptible to the denaturing constituents of the ClearSee solution compared with the other fluorescent proteins and that in order to preserve its fluorescently competent state it needs to be crosslinked by formaldehyde more extensively. However, we have not been able to achieve a sufficient degree of fixation for mNeonGreen under the conditions tested here.

Because stable transformation of rice is a time‐consuming process, heterologous expression systems or protoplast assays are used to test the function of rice proteins in a time‐efficient manner. Microparticle bombardment for transient transformation of cells in intact rice tissues such as leaves or roots is a useful alternative to these techniques. The preparation of microparticles and the bombardment of plant tissue can be performed in a short amount of time and protein accumulation is detectable in a relatively high number of cells only 24 hr after bombardment, making this a fast and efficient transformation method. In addition, it ensures that protein function is analyzed in the relevant cell and tissue context. Microparticle bombardment is not only suitable for analyzing processes such as the subcellular localization of proteins in different cell types of rice, but can also be used to study protein–protein interactions or the spatial regulation of gene expression. Together with a high‐throughput cloning technique such as Golden Gate (Engler et al., 2009; Patron et al., 2015; Weber et al., 2011), the findings reported here should facilitate molecular and cell biology analysis in rice, for example by allowing the rapid testing of gene expression vectors prior to stable transformation, analysis of protein‐transcription factor binding through transactivation assays, analysis of protein–protein interactions by bimolecular fluorescence complementation, and analysis of subcellular targeting of proteins.

## CONFLICT OF INTEREST

The authors declare no conflict of interest.

## AUTHOR CONTRIBUTIONS

J.M.H., L.H.L., and S.E. designed the experimental work. L.H.L. and S.E. cloned expression vectors for transient and stable transformation, respectively. N.W. carried out stable transformation of rice. L.H.L. performed microparticle bombardment experiments. L.H.L., S.E., and N.W. performed the microscopy. L.H.L., S.E., and J.M.H. wrote the manuscript. All authors revised the manuscript and approved the final version.
