# Supplementary material for: Fluorescent reporters for functional analysis in rice leaves
Source: Plant Direct. 2020 Feb 11;4(2):e00188. doi: 10.1002/pld3.188 (PMC7011658; doi:10.1002/pld3.188)
Supplement: Supplementary file 1 [file PLD3-4-e00188-s001.pdf]

Fluorescent reporters for functional analysis in rice leaves

Leonie H. Luginbuehl[†], Sherif El-Sharnouby[†], Na Wang, and Julian M. Hibberd[*]

Department of Plant Sciences, University of Cambridge, Downing Street, Cambridge CB2 3EA, United Kingdom.

[†]These authors contributed equally to this work.

*Corresponding author.

LHL - lhl28@cam.ac.uk

SE - se266@cam.ac.uk

NW - nw375@cam.ac.uk

JMH - jmh65@cam.ac.uk

Supplemental File 1

Supplemental File 1 contains five supplemental figures and one supplemental table.

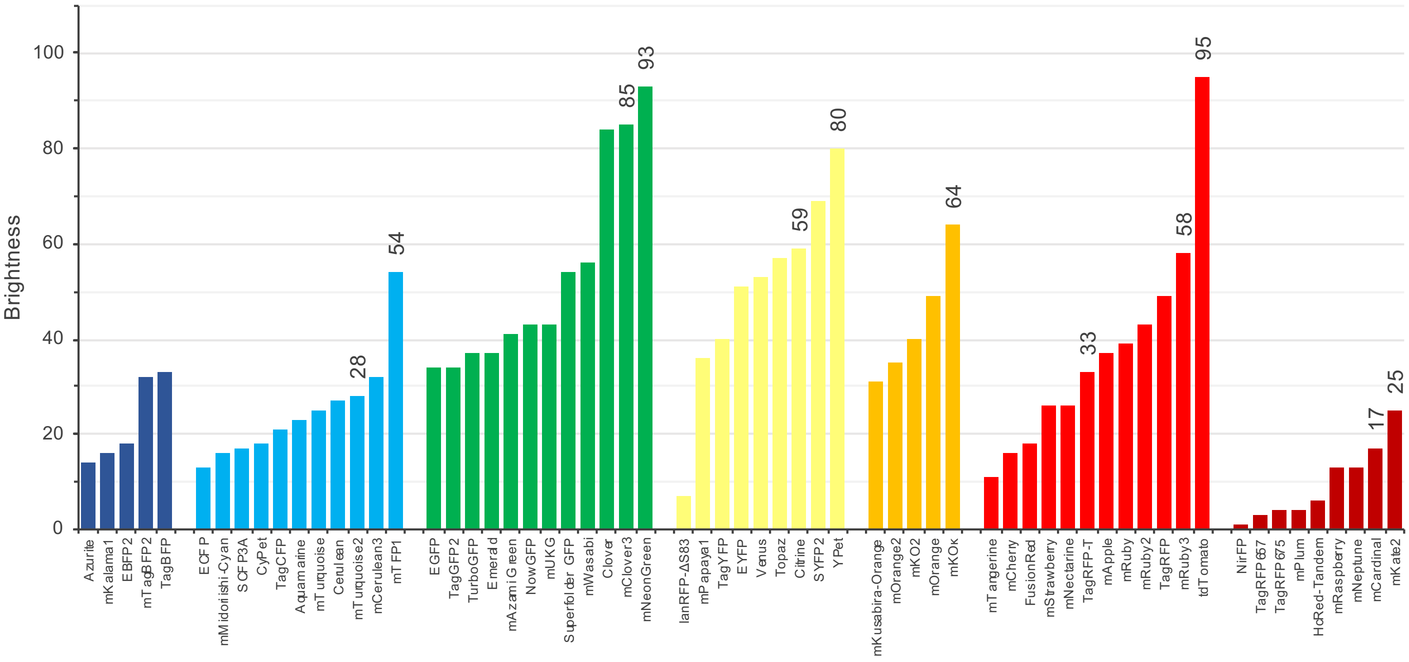

Supplemental Figure 1: Fluorescent proteins chosen as candidate reporters to test in rice. The twelve fluorescent proteins selected for screening are annotated with a value representing their brightness (product of extinction coefficient and quantum yield). From left to right: blue, cyan, green, yellow, orange, red and far red fluorescent proteins. The displayed set of proteins was obtained from (http://www.fpvis.org/FP.html), except for tdTomato, which was missing from the database.

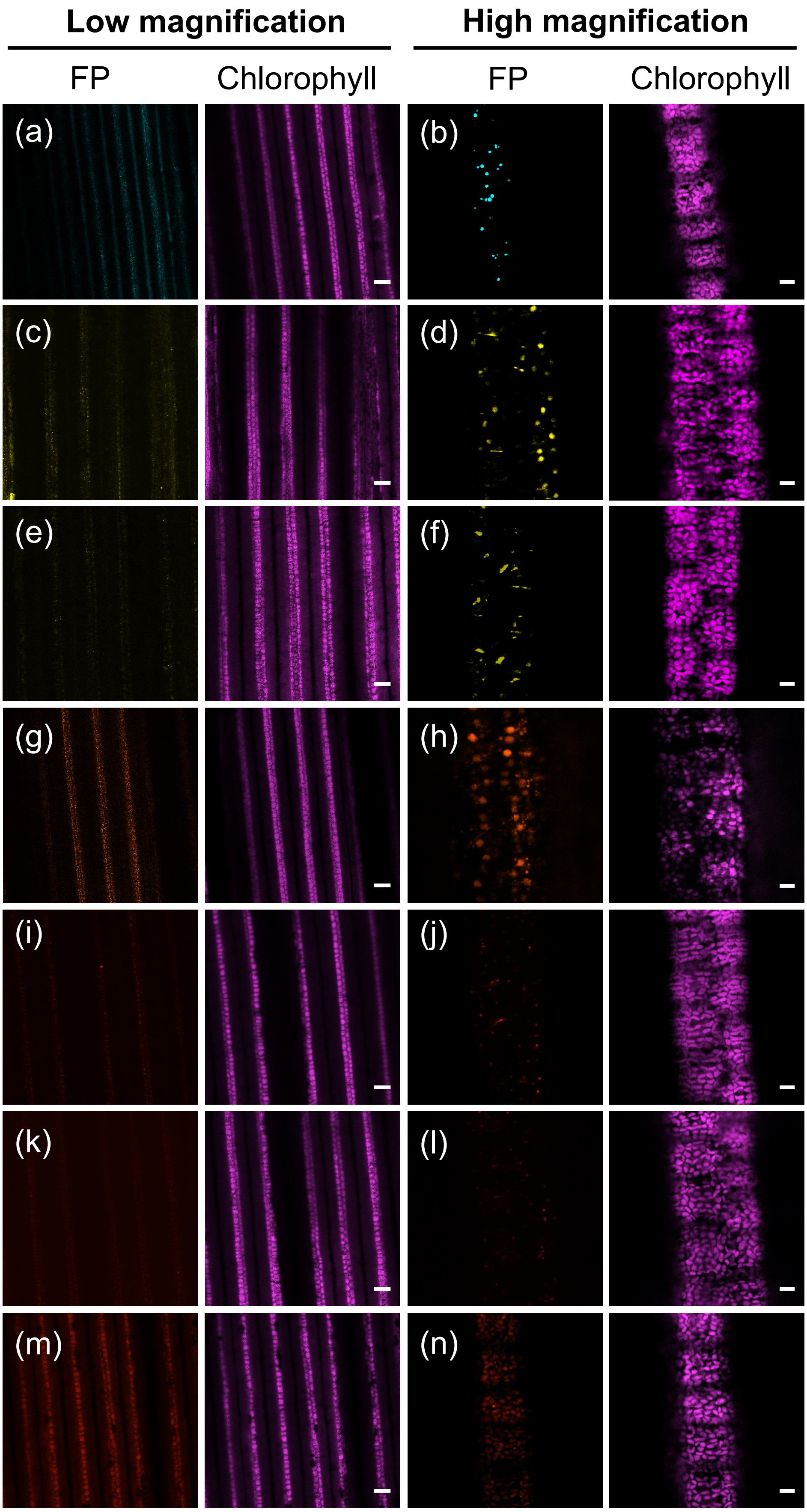

Supplemental Figure 2: Undetectable and poorly detectable fluorescent proteins expressed in mesophyll cells of rice leaf blades from stably transformed T_0 plants. (a,b) Leaves expressing *ZmPEPC$_{pro}$:mTFP1-NLS.* (c,d) Leaves expressing *ZmPEPC$_{pro}$:mCitrine-NLS.* (e,f) Leaves expressing *ZmPEPC$_{pro}$:mYPet-NLS.* (g,h) Leaves expressing *ZmPEPC$_{pro}$:TagRFPT-NLS.* (i,j) Leaves expressing *ZmPEPC$_{pro}$:mRuby3-NLS.* (k,l) Leaves expressing *ZmPEPC$_{pro}$:mKate2-NLS.* (m,n) Leaves expressing *ZmPEPC$_{pro}$:mCardinal-NLS*. FP; fluorescent protein. Scale bars represent 100 μm and 10 μm for low and high magnification images, respectively.

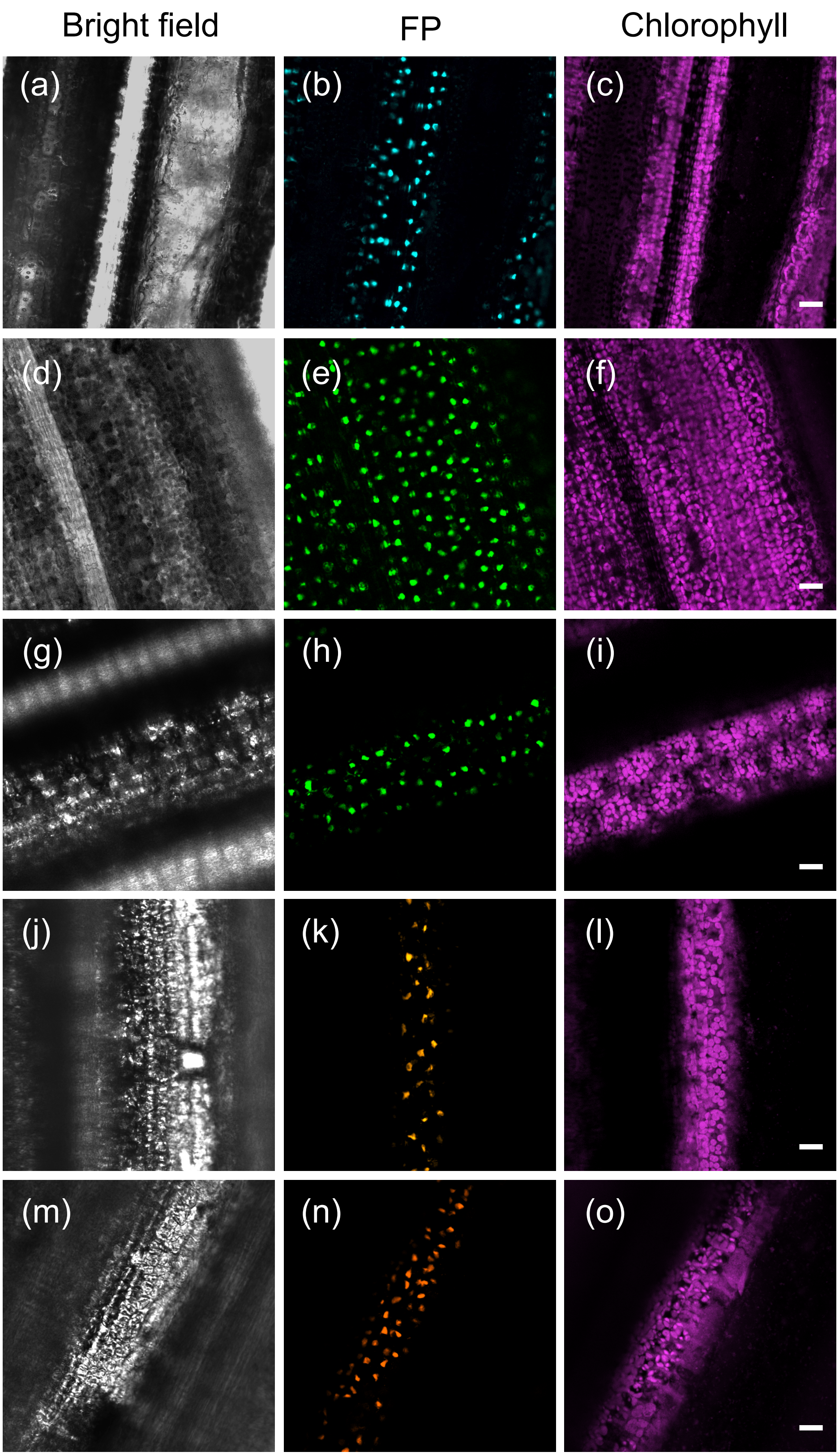

Supplemental Figure 3: Fluorescent proteins expressed in mesophyll cells of rice leaf blades from stably transformed T_1 plants. (a-c) Leaves expressing *ZmPEPC$_{pro}$:mTurquoise2-NLS.* (d-f) Leaves expressing *ZmPEPC$_{pro}$:mNeonGreen-NLS.* (g-i) Leaves expressing *ZmPEPC$_{pro}$:mClover3-NLS.* (j-l) Leaves expressing *ZmPEPC$_{pro}$:mKOκ-NLS.* (m-o) Leaves expressing *ZmPEPC$_{pro}$:tdTomato-NLS*. FP; fluorescent protein. Scale bars represent 20 μm.

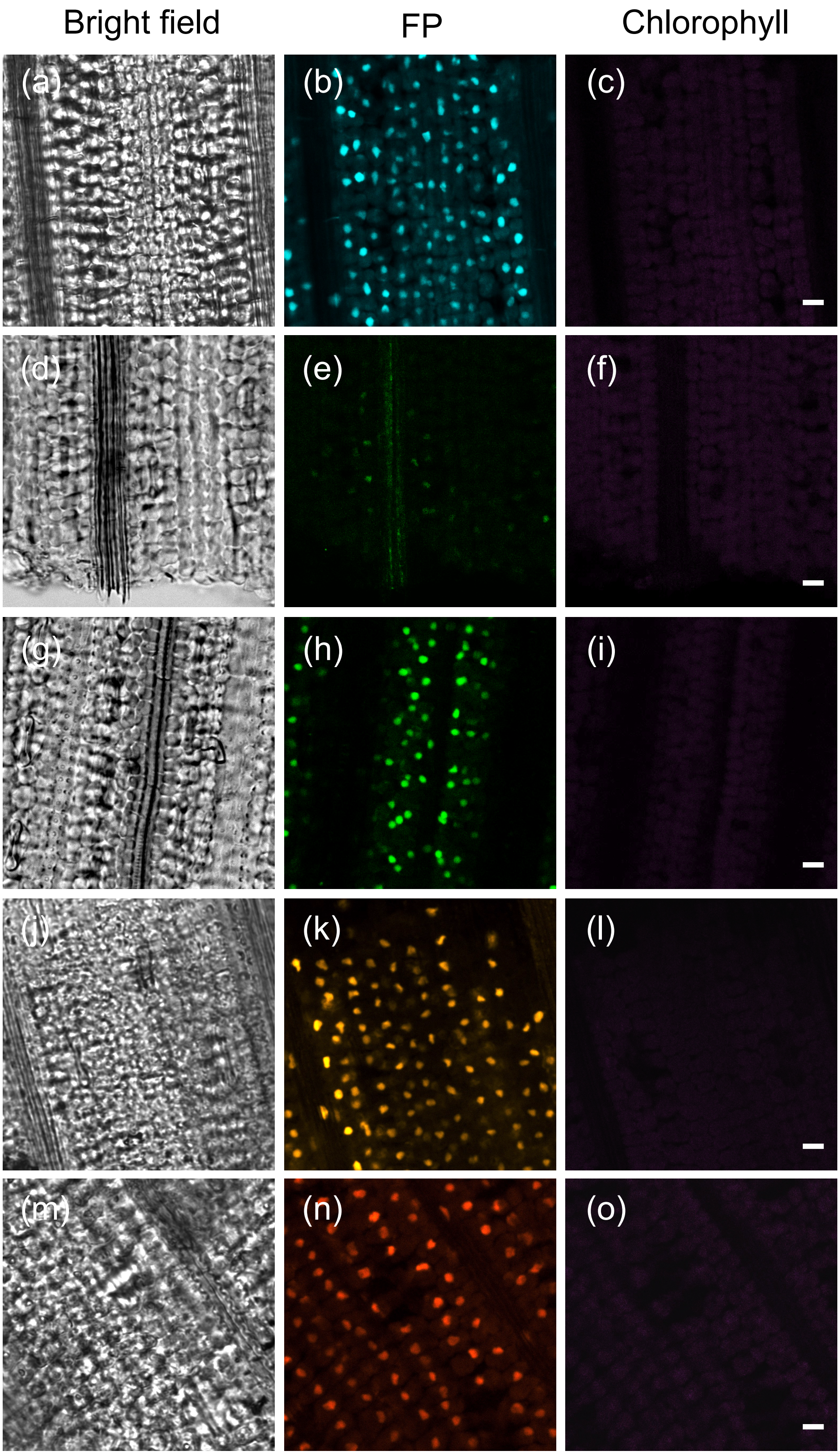

Supplemental Figure 4: Fluorescent proteins expressed in mesophyll cells of ClearSee-treated rice leaf blades from stably transformed plants. (a-c) Leaves expressing *ZmPEPC$_{pro}$:mTurquoise2-NLS.* (d-f) Leaves expressing *ZmPEPC$_{pro}$:mNeonGreen-NLS.* (g-i) Leaves expressing *ZmPEPC$_{pro}$:mClover3-NLS.* (j-l) Leaves expressing *ZmPEPC$_{pro}$:mKOκ-NLS.* (m-o) Leaves expressing *ZmPEPC$_{pro}$:tdTomato-NLS*. FP; fluorescent protein. Scale bars represent 10 μm.

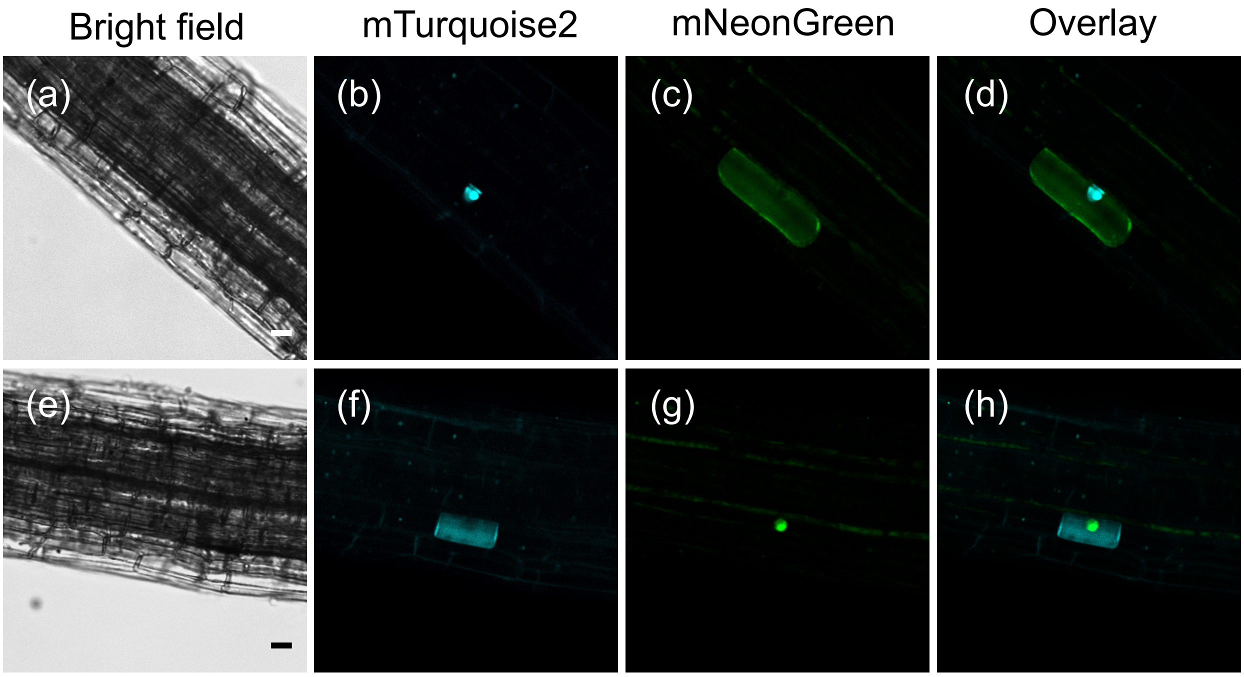

Supplemental Figure 5: Fluorescent proteins targeted to different cell compartments in transiently transformed rice root cells. (a-d) Root cell transformed with a construct expressing nuclear-localized mTurquoise2 and plasma membrane-localized mNeonGreen. (e-h) Root cell transformed with a construct expressing plasma membrane-localized mTurquoise2 and nuclear-localized mNeonGreen. Scale bars represent 10 μm.

Supplemental Table 2: Screen of stably transformed T_0 plants expressing different fluorescent proteins. Numbers indicate independent T_0 plants for each fluorescent protein where nuclear fluorescence signal was or was not detected.

Reporter	Nuclear signal detected	Not detected	Notes
mTurquoise2	5 strong / 3 weak	1	Clear nuclear signal, relatively little signal from autofluorescent structures
mNeonGreen	5 strong / 4 weak	0	Clear nuclear signal, substantial signal from autofluorescent structures
mClover3	7 strong	2	
mKOκ	7 strong	0	
tdTomato	4 strong / 3 weak	0	
TagRFP-T	3 strong / 3 weak	0	Non-robust nuclear signal, substantial signal from autofluorescent structures
mCitrine	2 weak	3	Weak nuclear signal for very few plants, substantial signal from autofluorescent structures
mYPet	1 weak	4	
mTFP1	none detected	6	No nuclear signal detected, autofluorescence signal present
mRuby3	none detected	7	
mKate2	none detected	7	
mCardinal	none detected	5	
