# Supplementary material for: Fluorescent reporters for functional analysis in rice leaves
Source: Plant Direct. 2020 Feb 11;4(2):e00188. doi: 10.1002/pld3.188 (PMC7011658; doi:10.1002/pld3.188)
Supplement: Supplementary file 3 [file PLD3-4-e00188-s003.pdf]

Dear Editor,
Thank you very much for handling the manuscript, along with your comments and those of the Reviewers. We have modified the manuscript to address these points. We hope that the work is now acceptable for publication in *Plant Direct*.

Reviewer #1

1. Line 338. The authors stated that "the detergent we used during fixation of rice, which was not used during fixation of Arabidopsis, adversely affects protein stability or fluorescence of mClover3 and mNeonGreen." I wonder if there is a possibility that the stability of these tags is time dependent rather than detergent sensitive, as the clearing took at least few weeks. Is it possible that after removing epidermal and subtending mesophyll cell layers and clearing with ClearSee, mClover3 and mNeonGreen still maintain the signal for certain time?

We thank the reviewer for this suggestion. To address it, we have performed additional experiments in an attempt to get ClearSee working with mClover3 and mNeonGreen. Specifically, we tested fixation with and without Silwet L-77, with and without scraping one or both sides of the leaf (to potentially improve fixation efficiency), and for different amounts of time (ranging from 45 min to overnight). We also tested whether clearing with ClearSee for 3-4 days (instead of 4 weeks) is sufficient. We found that scraping the leaf prior to fixation (with formaldehyde solution containing Silwet L-77) and fixing for at least 1.5 hr (ideally 3 hr) led to a drastic improvement in the detectability of mClover3. From these results we conclude that mClover3 requires more extensive fixation than mTurquoise2, mKOκ and tdTomato. We have updated the Results and Materials and Methods sections of the paper accordingly (pages 7&8, lines 216-223; page 10, lines 290-296). However, for mNeonGreen, detection only improved slightly and fixation times longer than 3 hr did not make a difference (we have clarified this as well in the text). Addressing this suggestion has brought to our attention an important point: we have been able to confirm that clearing for as little as 4 days is sufficient for successful clearing and fluorescent protein visualization and treatments of 4 weeks are unnecessary for rice leaf samples, similar to what was observed for Arabidopsis leaves in the original ClearSee publication. We have highlighted this in the manuscript (page 8, lines 227-229).

2. I wonder if authors considered clearing the removed cell layers or bigger segments of the leave blade and if that improved visualization of weakly and hardly detectable fluorescent proteins, as well as removed most part of autofluorescence? Would this speed up the clearing and preserve the fluorescent proteins?

If we understand correctly, the reviewer is referring to the undetectable (mTFP1, mRuby3, mKate2, mCardinal) and poorly detectable (mCitrine, mYPet, TagRFP-T) fluorescent proteins and asking if we tried detecting them in conjunction with reducing autofluorescence by ClearSee. We thank the reviewer for this suggestion, however we think that our inability to detect these particular fluorescent proteins is due to inherent problems with the expression and/or stability of these proteins rather than autofluorescence interfering with detection. Our reasoning is based on our observations that nuclei are generally minimally autofluorescent and so even in the presence of strongly autofluorescent structures in the cytoplasm or cell wall/extracellular space, if there is stable expression of the fluorescent proteins in the nucleus then we would expect to detect them regardless of their expression level and despite the presence of autofluorescent structures. We of course cannot rule out the possibility that the substantial signal from autofluorescent structures (especially for mCitrine, mYPet, TagRFP-T and mRuby3) could distract from our ability to find and image weakly expressing nuclei. We have made this point in the text (page 12, lines 335-341).

3. Line 167. Correct "Seven µl"

When starting a sentence with a number, we preferred the convention of spelling it out as a word. We will of course follow the instructions from the copy editors in this regard, and are more than willing to change to the number if required.

4. Unify the writing of "mKOk" throughout the text

We apologize for the inconsistency, and have now addressed this point. (We did not highlight each mKOκ in yellow in the text.)

Reviewer #2

1. *Introduction – Line 52 "have" should be "has"*

We have re-read this, and believe that our grammar was correct. We were referring to both "the development of a wide range of colour variants based on GFP" and "the discovery of new fluorescent proteins" having enabled multicolour imaging. However, we also agree that the sentence could have been better phrased to make this clear. We have now done so (page 3, lines 52-56).

2. *Introduction – The sentence across lines 70-72 is unclear. A "limited number" of proteins have been used in rice; is this solely the three listed in the sentence? How many proteins is "limited"?*

We agree that this sentence was confusing. We have modified the text to address this (page 3, lines 78-81).

3. *Introduction – Line 83 "are" should be "is"*

Thank you. We have modified the text to make the sentence grammatically correct (page 4, lines 89-92).

4. *Materials and Methods – The authors list a variety of parameters that were used for selecting fluorescent proteins for testing; It would be of interest to have a supplementary table that describes the various values ascribed to the fluorescent proteins for each of the parameters listed (lines 104-106).*

Thank you for this point. We agree. To avoid generating redundant information, we have now included reference to the publicly accessible fluorescent protein database containing this information (https://www.fpbase.org - page 5, line 119).

5. *Materials and Methods – Supplemental Figure 1—where was TdTomato taken from?*

We apologize for the lack of clarity. The database (http://www.fpvis.org/FP.html) from which the proteins in Supplemental Figure 1 were obtained does not contain tdTomato. However, because we were aware that tdTomato is one of the brightest existing proteins, we wished to include it in our analysis. We therefore obtained information regarding its optical parameters from Shaner et al., (2004). The http://www.fpvis.org/FP.html database has recently been upgraded (https://www.fpbase.org) and now contains a more extensive and growing collection of fluorescent proteins including tdTomato. We have provided reference to this database in the Materials and Methods (page 5, line 119).

6. *Materials and Methods – The authors provide a comprehensive outline of the sources of the fluorescent protein sequences in lines 109 onwards. However, they then mention that the various sequences have been codon optimized, domesticated, etc. Can the authors please make these sequences available, either through vector deposition on Addgene (which would be optimal) or in a supplementary file including all of the gene sequences used in the paper. As the authors mention themselves (lines 315-317), it's possible that codon optimization may affect protein expression or stability. In particular, given the main utility of this paper as a resource for other scientists using fluorescent reporters in rice, it would be very beneficial if the successfully tested constructs, at a minimum, could be made available through Addgene.*

We fully agree with the reviewer. We have made the Level 0 vectors for the robustly detectable fluorescent proteins (mTurquoise2, mNeonGreen, mClover3, mKOκ and tdTomato) available through Addgene (page 5, lines 143-145). We have also included an additional supplementary file containing the sequence of each fluorescent protein used in our study. This has also been clarified in the text (page 5, lines 137-138).

7. *Materials and Methods – Line 164, what age roots were used?*

We used roots from 12-day old seedlings for bombardment. We have clarified this in the Materials and Methods section (page 6, line 183).

8. Results – Both results sections start with a substantial portion of introductory material (lines 217-223 and 273-283); I would suggest that this information is integrated into the introduction, leaving only the results in the results section.
We thank the reviewer for this point, and have removed this information from the results sections.

9. Results – I would suggest that Supplementary Figure 2 should be made into a main figure, given that the discussion of the figure takes up a substantial portion of the results.
We agree with the reviewer and have now included the data from Supplementary Figure 2 as a main figure. These findings are now part of Figure 1 (page 20, lines 586-598).

10. Results – The reference to Supplementary Figure 3 is ambiguous, and combined with the figure title (which is identical to Figure 1) it took me a few moments to figure out that SF3 was showing the fluorescent reporters which didn't work. Please make this more explicit in the text, and make the figure titles more informative.
We agree with the reviewer and have addressed this ambiguity in the text and the figure titles.

11. Discussion – I would suggest that you add some more details to the statement in line 308. Is rice important economically? As a source of calories? Please expand on this, and include a reference.
Thank you, we have added two citations relevant to the importance of rice as a global crop (page 12, lines 324-325).

Reviewer #3
1. Having the excitation and emission wavelengths of the fluorescent proteins used (in the confocal imaging part of the MandM) will make it neater and more visible for future references.
Thank you, we have included reference to a fluorescent protein database containing excitation and emission spectra for the 12 fluorescent proteins used in our study. We have also included the precise excitation and emission parameters that we used for imaging (page 8, lines 232-240).

2. Line 198 (plus line 265): The fixation is actually made in formaldehyde not paraformaldehyde since paraformaldehyde changes its chemistry in solution and becomes formaldehyde... For more information, please check the following paper: https://www.ncbi.nlm.nih.gov/pubmed/12769269
We particularly thank the reviewer for drawing this to our attention. We have corrected the erroneous use of paraformaldehyde in the text (page 7, lines 216-217).

3. Figure 2 (legend) there is only one scale bar
Thank you, we have indicated in the Figure legend that all images were taken at the same magnification.

4. Make sure that all the species Latin names in the "references" are written in italic.
Thank you. We have corrected these (not highlighted in text).

Once again, we are grateful to the careful analysis of all Reviewers. We hope that we have addressed the points adequately. On behalf of all authors.

Yours sincerely,

Julian Hibberd
